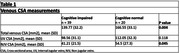# Alzheimer's disease subjects have smaller non‐jugular venous drainage system compared to cognitive normal population

**DOI:** 10.1002/alz.093759

**Published:** 2025-01-09

**Authors:** Keshet Prado, Vadim Khasminsky, Ophir Keret, Felix Beninger, Ilan Goldberg, Eitan Auriel, Amir Glik

**Affiliations:** ^1^ Tel Aviv University, Tel Aviv Israel; ^2^ Rabin Medical Center, Petach Tikva Israel; ^3^ Tel Aviv university, Tel aviv Israel; ^4^ Department of Neurology, Rabin Medical Center, Beilinson Hospital, Petach Tikva Israel; ^5^ Rabin medical center, Petach Tikva Israel

## Abstract

**Background:**

The Internal Jugular Veins (IJVs) and the non‐jugular veins (NJVs) are two pathways responsible for intracranial blood drainage. The NJVs are usually the less prominent drainage system and have been demonstrated to become smaller during aging. This phenomenon may indicate less affective venues drainage and hence less CNS’s waste products clearance as we age. One of the pathological hallmarks in Alzheimer’s disease (AD) is amyloid betta42 (AB42) accumulation. AB42 accumulation may be the result of overproduction or clearance impairment. NJVs narrowing during aging may cause AB42 accumulation due to clearance impairment and hence plays a role in AD pathology. Our aim was to compare the venous cross‐sectional area (CSA) in AD vs. cognitive normal (CN) population.

**Method:**

Cognitive impaired patients included AD and mild cognitive impairment (MCI) patients that were recruited prospectively as part of the DIASPORA Horizon‐2020 MesCobrad Project. All patients performed comprehensive neurocognitive evaluation and Brain MRI including Time‐of‐flight sequence for venous demonstration. Venous systems CSA were measured at the jugular foramen level and were done by senior neuroradiologist. CN were defined as subjects without CNS’s known pathology. CN Measurements were taken from a previous study of our group. T‐test was used to compare between groups.

**Result:**

A total of 39 Cognitive impaired patients and 20 CN were included in the study. Mean ages of 68 years (SD, 5.4) and 66 years (SD, 5.5), respectively. The CSA of the IJVs was similar between groups. However, NJVs CSA was significantly smaller in cognitive impaired patients (mean CSA=41.21 mm2 (SD,21.52) vs. CSA=54.5 mm2 (SD,27.3) P=0.045). Table 1.

**Conclusion:**

NJVs are significantly smaller in cognitive impaired patients compared to CN population. NJVs narrowing in AD patients may cause impaired venous drainage hence causing AB42 accumulation in this population. New treatment options for AD patients may rise if larger studies replicate our findings.